# A History of Medical Libraries and Medical Librarianship: From John Shaw Billings to the Digital Era

**DOI:** 10.5195/jmla.2022.1324

**Published:** 2022-04-01

**Authors:** Eleanor Shanklin Truex

**Affiliations:** 1 eleanor.truex@amitahealth.org, Medical Librarian, Chicago Metro Region, Amita Saint Joseph Hospital, Saints Mary and Elizabeth Medical Center, Chicago, IL

This comprehensive tome is the work of the Kronenfelds, coauthors who now turn their attention to the medical information sciences field, which Michael has seen dramatically change since his beginnings in it circa 1975. This book comes not a moment too soon, as stated in its preface:

The purpose of this book is not only to make this history available to the profession's practitioners, but also to provide context as medical librarians and libraries enter a new age in their history as the digital information environment has undercut the medical library's previous role as the depository of the print-based KBI/Information base [p. xvii].

When my predecessor at our hospital started her medical library career in 1974, she asked the nuns for a then innovative fax machine; the library has never looked back.

I share this to demonstrate that while remaining a service profession, much has changed in the medical library world in the past fifty years, never mind the past 180 when the army surgeon general's library was founded.

Swift, relentless change is not new; it remains the constant in medicine and all fields associated with it. How and if today's medical librarians and their libraries endure these transformations is anyone's guess, but the Kronenfelds' book is here to help. This book is not a guide to the newest bell or whistle for the medical librarianship role; this book stands as a helpmeet based on that old truism: we can't know where we're going unless we know where we've been. Consider this book to be a contextual natural history for medical librarianship.

What struck me while reading this history was how young medical librarianship actually is, and how much of the growing has been both despite and because of societal upheaval, war, technological revolutions, and changes to our health care system. The National Library of Medicine only formally came into being in 1956; that's sixty-five short years for such a complicated and densely packed history.

The ways medical librarians, either academic or hospital-based, have risen repeatedly to the challenges presented them pervade this work. Michael was there for much of this rich history, and the book benefits from his engagement and experience. It is one thing to read a chronicle; it is another to read an account from someone who has been active in helping create that history over the past forty-five years.

The book is organized into seven eras, but there is overlap between them, so the reader starts to see how the “dots,” such as the evolution of MEDLINE from MEDLARS, are connected. Topics like these are revisited across the eras so context is revealed. The timeline at the end of the book is invaluable; another timeline of the development and the evolution of Index Medicus to its current iteration would have provided an encapsulation of our essential service across the decades. Acronyms abound, but thankfully there is a handy list to help any reader swimming in that alphabet soup. Lest the reader think this work is only retrospective, the contemporary portion (the Emerging Era of the Digital Health Science Library) supplies a robust exploration of the digital age and the subsequent displacement of traditional print media and collection approaches.

Finally, the appendixes range from past milestones to a thorough exploration of the current state of play, with the timelines as well as the bibliography providing a wealth of information for the curious reader.

This work is exhaustive in its scope but is also meticulously researched. It is a solid choice for a medical librarianship graduate-level class if these programs have the space in their packed curricula.

This book will prove absorbing to any aspiring or actual medical librarian; the content is essentially a ringside seat to America from 1836 to the modern day as seen through the lens of medical librarianship with its ties to medical and societal change.

This reviewer takes away a new appreciation for the ground covered by her profession, but also a respect for the formidable challenges that lay ahead. It is clearly a work of great meaning for both authors and is a welcome addition to the other chronicles that explore our world.

